# Robust optimization for multi-project scheduling via the critical chain method

**DOI:** 10.1371/journal.pone.0336350

**Published:** 2025-11-13

**Authors:** Min Tian, Xiaomei Li

**Affiliations:** 1 School of economics and management, Xidian university, Xi’an, China; 2 Guangzhou Institute of Technology, Xidian University, Guangzhou, China; 3 School of Management, Xi’an Polytechnic University, Xi’an, China; Shanghai Jiao Tong University - Xuhui Campus, CHINA

## Abstract

The critical chain method is often used to improve robustness in single-project scheduling, but there are two challenges when applying it to multi-project scheduling. First, the existing robustness measure focuses on time elasticity within sub-projects but neglects elasticity across sub-projects, making it difficult to balance drum resource requirements. Second, the differential evolution (DE) algorithm is adopted to solve this problem, but continuous evolutionary operators have limited flexibility, leading to numerous transformations between the continuous solution space and the discrete problem space. Therefore, we adjust the critical chain multi-project scheduling model by incorporating the drum buffer and the capacity constraint buffer and propose a robustness measure that considers both time elasticity within and among sub-projects. Meanwhile, we design an enhanced discrete DE algorithm, which not only discretizes the encoding–decoding strategy and evolutionary operators but also uses a hill-climbing algorithm to enhance local search. Experiments are conducted to verify the effectiveness of the robustness measure and the algorithm. The results indicate that, averaged over the eight instances, the enhanced discrete DE algorithm achieves an improvement of more than 3.3% in robustness compared with the overall mean of the benchmark algorithms. Furthermore, our robustness measure strengthens the stability of the scheduling plan and reduces buffer consumption and overflow during multi-project scheduling.

## 1. Introduction

Project scheduling is inherently subject to pervasive uncertainties that frequently cause fluctuations in activity durations. Such variability can significantly undermine schedule stability. A large-scale study by Szwarcfiter et al. [1], involving 50,000 projects across 1,000 organizations, reported that over 60% of projects suffer delays due to uncertainty. Furthermore, nearly 90% of projects are executed in multi-project environments [2], where, compared with single-project settings, competition for the shared drum resource across sub-projects further exacerbates delay risk. Robust scheduling aims to address these challenges through either proactive planning or reactive adjustments, and empirical evidence indicates that proactive approaches generally outperform reactive ones [3]. Accordingly, this study adopts a proactive planning approach to optimize robust scheduling in multi-project environments.

Proactive planning anticipates uncertainty, thereby enabling the design of schedules with built-in resilience [4]. Because activity slack depends on sequencing, initial resequencing can improve robustness; however, limited slack constrains this approach’s effectiveness. To further enhance time elasticity, time buffers can be inserted in two ways. In decentralized buffer insertion, buffers are assigned to individual activities, increasing time elasticity but prolonging the overall project duration [5]. The centralized approach—the Critical Chain Method (CCM) [6]—reduces embedded safety time in activity estimates to mitigate Student Syndrome and Parkinson’s Law, and places buffers at chain termini in the scheduling network, thereby achieving a better balance between time elasticity and project duration. In this study, we employ CCM to further improve the schedule’s time elasticity, which, however, introduces challenges for the design of robustness measures and the development of solution algorithms.

When buffer insertion is not considered, activity slack is often used to measure a schedule’s time elasticity—for example, total activity slack [7] and weighted slack [8], among others. However, once buffers are introduced—which also affect the schedule’s time elasticity—the design of robustness measures becomes more complex. For instance, the CCM inserts a project buffer (PB) and feeding buffers (FBs) within a single project. Accordingly, some studies have developed robustness measures based on the time elasticity created by these buffers [9]. In multi-project environments, conflicts over the drum resource are a primary cause of schedule delays, and robustness measures that ignore time elasticity across sub-projects are not applicable. Following Newbold’s [10] theory, additional buffers—drum buffers (DBs) and capacity constraint buffers (CCBs)—are introduced to absorb delays in drum resource availability across sub-projects. Yet drum resource requirements vary across sub-projects, and designing robustness measures that account for time elasticity both within and across projects becomes more challenging.

Meanwhile, solving the multi-project scheduling problem is NP-hard. Both exact and heuristic algorithms have been applied to this problem [11,12]. Because exact methods scale poorly with problem size, heuristics are more widely used—such as Genetic Algorithm [13], Particle Swarm Optimization [14], and Differential Evolution (DE) [15]. Compared with other heuristics, DE is relatively simple and requires fewer parameters, so we adopt it in this study. However, existing DE algorithms operate in a continuous search space, whereas multi-project scheduling is inherently discrete. This necessitates frequent mappings between discrete problem and continuous solution spaces, which reduces computational efficiency and restricts search flexibility. Moreover, incorporating CCM introduces buffers into the chain structure, expanding variation in time elasticity within and across projects, thereby increasing the computational complexity of our problem.

In summary, a critical chain resource-constrained multi-project scheduling model is developed that incorporates the buffers and chain structures of CCM. Additionally, a robustness measure is designed as the optimization objective, capturing time elasticity both within and across sub-projects and accounting for different buffer types. An enhanced discrete DE algorithm is proposed to solve the model; it discretizes the encoding–decoding scheme and the evolutionary operators, and employs hierarchical neighborhood optimization to improve local search. Finally, computational experiments are conducted to validate the effectiveness of the robustness measure and algorithm.

The remainder of this paper is organized as follows: Section 2 reviews the relevant literature. Section 3 formulates the model and introduces a robustness measure. Section 4 develops an enhanced discrete DE algorithm to solve the model. Section 5 reports computational experiments to validate the effectiveness of the proposed robustness measure and algorithm. Section 6 concludes and discusses key findings.

## 2. Related work

### 2.1. Robustness measures in critical chain project scheduling

In problem formulations, robustness measures are typically employed as optimization objectives to quantify a schedule’s time elasticity. Historically, activity slack under different processing sequences was used to assess plan robustness. For example, Khemakhem and Chtourou [16] designed robustness measures based on activity slack to describe schedule stability in the resource-constrained project scheduling problem, dividing them into weight-based, path-based, and dispersion-based categories. Then, Mahalleh et al. [7] used the sum of activity slack as the robust objective for the resource-constrained project scheduling problem, assuming equal weights for all activities.

In order to further improve the time elasticity of the schedule, buffers are inserted into it. For example, Ghoddousi et al. [8] and Zheng and He [17] applied the decentralized buffer insertion method in robust scheduling and adopted weighted slack as the robust objective for the resource-constrained project scheduling problem. Moreover, Wang et al. [18] applied the CCM to sub-projects in a multi-project scheduling problem. They proposed a critical chain resource-constrained multi-project scheduling model to improve the robustness of the multi-project schedule, in which the average slack and minimum slack are used as robustness measures. In this scheme, the activity with the minimum slack has the maximum weight, while the others have equal weights.

Moreover, Hazır et al. [9] examined CCM in a multi-mode resource-constrained project scheduling problem, and compared a PB-related measure with eight robustness measures, including average slack, weighted slack, slack utility function, slack dispersion, and the percentage of potentially critical activities, among others. The effectiveness of these robustness measures was verified through Monte Carlo simulation experiments, and the results indicated that the PB-related measure outperformed other measures in terms of its correlation with on-time project delivery.

Table 1 summarizes the authors, problem types, and robustness measures based on the above literature. Overall, most studies primarily focus on time elasticity within single project settings. However, in multi-project scheduling problems, time elasticity also arises across projects, making these studies unsuitable for our problem.

**Table 1:** Summary of robustness measures in project scheduling.

Authors (year)	Problem Type	Robustness measure(s)
Khemakhem and Chtourou (2013)	resource-constrained project scheduling	activity-slack-based measures
Mahalleh et al. (2017)	resource-constrained project scheduling	sum of activity-slack-based measures with equal weights
Ghoddousi et al. (2016)	resource-constrained project scheduling	weighted activity-slack-based measure
Zheng and He (2017)	resource-constrained project scheduling	weighted activity-slack-based measure
Wang et al. (2014)	critical chain resource-constrained multi-project scheduling	average and minimum activity-slack-based measures
Hazır et al. (2010)	multi-mode resource-constrained project scheduling	buffer-related measure and eight activity-slack-based measures

### 2.2. DE algorithm for scheduling problems

The DE algorithm was first proposed by Storn and Price [19]. Because of its simple implementation and fast convergence, this algorithm has received extensive attention. Over the past decades, the DE algorithm has been widely used to solve scheduling problems. It was initially applied to job shop scheduling. For example, Han et al. [20] applied the DE algorithm to a hybrid flow shop scheduling problem and designed mutation, crossover, and selection operators in the continuous solution space to search for optimal solutions. Xu and Wang [21] proposed an improved DE algorithm for the hybrid flow-shop scheduling problem, in which special matrices were used for encoding–decoding in the continuous solution space. Li et al. [22] applied an effective hybrid self-adaptive DE algorithm to a flexible job shop scheduling problem with outsourcing operations and job priority constraints. In this algorithm, chromosomes are encoded in the continuous solution space, and a heuristic strategy is employed to enhance the initial population.

Then, the DE algorithm was gradually applied to project scheduling. Peng and Huang [23] proposed a DE algorithm with an improved mutation strategy to solve a critical chain resource-constrained project scheduling problem. Specifically, two strategies were combined with the mutation operator in the continuous solution space to improve global exploration and convergence. Yan et al. [24] adopted two parallel mutation operators to improve the performance of the DE algorithm for solving the resource-constrained project scheduling problem. In their algorithm, two parallel mutation operators were used to improve the search capability in the continuous solution space. Van der Beek et al. [15] employed a hybrid differential evolution algorithm to solve the resource-constrained project scheduling problem with a flexible project structure, in which evolutionary operators were iterated in the continuous solution space and a one-to-one competitive survival strategy was used to improve solution quality.

Table 2 summarizes the authors, problem types, and DE variants in the above literature. Notably, differential evolution operators typically act in the continuous solution space, but our research problem is a discrete optimization problem, which leads to a significant conversion overhead during the search process.

**Table 2:** Applications of DE algorithm in scheduling problems.

Authors (year)	Problem Type	DE Variant
Storn and Price (1997)	general optimization	original DE
Han et al. (2009)	hybrid flow shop scheduling	traditional continuous DE
Xu and Wang (2011)	hybrid flow shop scheduling	continuous DE with special encoding–decoding matrices
Li et al. (2022)	flexible job shop scheduling	continuous DE with heuristic initialization
Peng and Huang (2014)	critical chain resource-constrained project scheduling problem	continuous DE with an improved mutation strategy
Yan et al. (2014)	resource-constrained project scheduling	continuous DE with two parallel mutation operators
Van der Beek et al. (2024)	resource-constrained project scheduling with flexible project structure	continuous DE with one-to-one competitive survival strategy

## 3. Formulation of the robust scheduling problem

### 3.1 Critical chain multi-project scheduling model

Assume there are N sub-projects in the multi-project, and each sub-project, denoted $P_{i}$, has $J_{i}$ activities. The parameter i represents a sub-project index, where i ranges from 0 to N−1. The parameter j represents an activity index, where j ranges from 0 to $J_{i}-\text{1}$. A multi-project, denoted P, is composed of N sub-projects, i.e., $P=\{P_{\text{0}},\ldots ,P_{N-\text{1}}\}$. Each sub-project includes activities with precedence constraints and resource requirements. Consider sub-project $P_{i}=\{a_{i\text{0}},\ldots ,a_{ij},\ldots ,a_{iJ_{i}-\text{1}}\}$ as an example to illustrate the precedence constraints among activities. There are $J_{i}$ activities in sub-project $P_{i}$. The first activity, $a_{i\text{0}}$, and the last activity, $a_{iJ_{i}-\text{1}}$, are dummy activities. We consider only renewable resources. Activities are not interrupted once they begin, and they may start only after their predecessor activities are completed. There is only one execution mode for each activity. We ignore transfer time between activities when resources are sufficient. The project deadline, activity durations, and resource requirements are predetermined positive integers. Sub-projects may be performed in parallel when resources are sufficient.

We further describe the variables to clarify the problem. In sub-project $P_{i}$, the durations and resource requirements of the dummy activities, $a_{i\text{0}}$ and $a_{iJ_{i}-\text{1}}$, are zero. The start time of the first activity, $a_{i\text{0}}$, is the start time of the sub-project. The finish time of the last activity, $a_{iJ_{i}-\text{1}}$, is also the finish time of the sub-project. The deadline of the sub-project is $D_{i}$. At time t, the set of ongoing activities in sub-project $P_{i}$ is $A_{it}$. The symbol $a_{ij}$ denotes an activity in this sub-project. The k-th resource requirement, total duration, most likely duration, and safety time of activity $a_{ij}$ are $r_{ijk}$, $t_{ij}$, $d_{ij}$, and $u_{ij}$, respectively. The process-precedence set and resource-precedence set of activity $a_{ij}$ are ${Pre}_{ij}$ and ${Prer}_{ij}$, respectively. There are at most $K_{l}$ types of local resources within sub-projects. For sub-project $P_{i}$, the total supply of the $k_{l}$ -th local resource $\left(\text{0}\leq k_{l}\leq K_{l}-\text{1}\right)$ is $R_{iK_{l}}$. Local resource usage is independent across sub-projects; therefore, there is no competition for local resources among them. There is one global resource (the drum resource) shared across sub-projects. The total supply of the drum resource is $R_{g}$, and its index is $K_{l}$.

We standardize both critical and non-critical paths within a sub-project as task chains to facilitate buffer calculations in a multi-project scheduling plan. An activity that uses the drum resource is referred to as a drum activity. In sub-project $P_{i}$, if $a_{ij}$ is a drum activity, its corresponding DB is denoted ${DB}_{ij}$. Let ${ac}_{i}$ denote a task chain in sub-project $P_{i}$; it consists of three activities, $a_{ij^{\prime }}$, $a_{il}$, and $a_{ij}$, located on the chain. Activity $a_{ij^{\prime }}$ is also a drum activity, positioned immediately before $a_{ij}$, whereas $a_{il}$ is a non-drum activity located between $a_{ij^{\prime }}$ and $a_{ij}$.

Let ${DA}_{i}$ denote the set of drum activities in sub-project $P_{i}$. Let ${DBA}_{i}$ denote the special-activity set—i.e., all activities used in the computation of drum buffers (DBs)—in sub-project $P_{i}$. Furthermore, ${CC}_{i}$ denotes the critical chain of sub-project $P_{i}$ with a project buffer ${PB}_{i}$, and ${NC}_{ihj}$ denotes a non-critical chain with a feeding buffer ${FB}_{ihj}$. Here, activity $a_{ij}$ is a critical activity on ${CC}_{i}$, and $a_{ih}$ is a non-critical activity that serves as a feeding node preceding $a_{ij}$.

Moreover, let $si^{\prime }$ denote the set of implemented sub-projects that have drum resource conflicts with the implementing sub-project $P_{i}$. Each sub-project in $si^{\prime }$ is indexed by $i^{\prime }$. The early drum activity set includes all drum activities that conflict with those in implemented sub-projects. The early drum activity set in sub-project $P_{i}$ is denoted ${ds}_{i}$ (where ${ds}_{i}\subseteq {DA}_{i}$). The late drum activity set includes all drum activities that conflict with those in the implementing sub-project. The late drum activity set corresponding to the implementing sub-project $P_{i}$ is denoted ${ds}_{i^{\prime }}$ (where ${ds}_{i^{\prime }}\subseteq {DA}_{i^{\prime }}$). Additionally, different sub-projects use the drum resource with different priorities, and the priority of sub-project $P_{i}$ is denoted $\text{priority}\left(P_{i}\right)$. The capacity constraint buffer (CCB) of sub-project $P_{i}$ is denoted ${CCB}_{i}$.

Based on the definitions above, we formulate the critical chain resource-constrained multi-project scheduling problem as follows:


f=𝐌𝐚𝐱(RM) (1)

$$s.t.u_{ij}=t_{ij}-d_{ij},i\in [\text{0},N-\text{1}],j\in \left[\text{0},J_{i}-\text{1}\right]$$ (2)

$$s_{ij}+d_{ij}\leq s_{ih},a_{ij}\in {Pre}_{ih}\cup {Prer}_{ih},j\neq J_{i}$$ (3)

$$\sum_{a_{ij}\in A_{it}}r_{ijk_{l}}\leq R_{ik_{l}},t\in \left[s_{i\text{0}},s_{iJ_{i}-\text{1}}\right],i\in [\text{0},N-\text{1}],j\in \left[\text{0},J_{i}-\text{1}\right],k_{l}\in \left[\text{0},K_{l}-\text{1}\right]$$ (4)

$$\sum_{i=\text{0}}^{N-\text{1}}\sum_{a_{ij}\in A_{it}}r_{ijk_{l}}\leq R_{g},t\in \left[s_{i\text{0}},s_{iJ_{i}-\text{1}}\right],j\in \left[\text{0},J_{i}-\text{1}\right]$$ (5)

$${DB}_{ij}=\sqrt{\sum_{\begin{array}{c} j^{\prime }\leq l<j\\ anda_{il}\in {ac}_{i} \end{array}}u_{il}^{\text{2}}},a_{ij^{\prime }}\in {DA}_{i},a_{ij}\in {DA}_{i},a_{ij^{\prime }}\in {ac}_{i},a_{ij}\in {ac}_{i}$$ (6)

$${FB}_{ihj}=\sqrt{\sum_{\begin{array}{c} a_{il}\in {NC}_{ihj}\\ anda_{il}\notin {DBA}_{i} \end{array}}u_{il}^{\text{2}}},a_{ij}\in {CC}_{i},a_{ih}\in {NC}_{ihj},a_{ih}\in {Pre}_{ij}\cup {Prer}_{ij}$$ (7)

$${PB}_{i}=\sqrt{\sum_{\begin{array}{c} a_{il}\in {CC}_{i}\\ anda_{il}\notin {DBA}_{i} \end{array}}u_{il}^{\text{2}}}$$ (8)

$${CCB}_{i}=\sqrt{\sum_{\begin{array}{c} i^{\prime }\in si^{\prime }\\ anda_{i^{\prime }l}\in {ds}_{i^{\prime }} \end{array}}u_{i^{\prime }l}^{\text{2}}}$$ (9)

$$s_{ih}+d_{ih}+{DB}_{ij}\leq s_{ij},a_{ij}\in {ac}_{i},a_{ih}\in {ac}_{i},a_{ih}\in {Pre}_{ij}\cup {Prer}_{ij}$$ (10)

$$s_{ih}+d_{ih}+{FB}_{ihj}\leq s_{ij},a_{ij}\in {CC}_{i},a_{ih}\in {NC}_{ihj},a_{ih}\in {Pre}_{ij}\cup {Prer}_{ij},$$ (11)

$${\text{max}}_{i^{\prime },h}\left(s_{i^{\prime }h}+d_{i^{\prime }h}\right)+{CCB}_{i}\leq {\text{min}}_{j}\left(s_{ij}\right)\;,P_{i^{\prime }}\in si^{\prime },a_{i^{\prime }h}\in {ds}_{i^{\prime }},a_{ij}\in {ds}_{i},\text{priority}\left(P_{i^{\prime }}\right)\geq \text{priority}\left(P_{i}\right)$$ (12)

Equation (1) represents the scheduling objective—to maximize robustness; its specific formulation is detailed in Section 3.2. Equation (2) defines the safety time of an activity as the difference between its total duration and its most likely duration. Equation (3) ensures that the start time of a successor activity is no earlier than the finish time of its predecessor. Equation (4) imposes the local resource constraints, ensuring that total usage at any time does not exceed the available supply within each sub-project. Equation (5) imposes the drum-resource constraint, requiring that total consumption across sub-projects does not exceed the available supply. Equation (6) calculates the DB on a task chain within a sub-project. Equation (7) computes the FB, excluding safety times already used in DB calculations. Equation (8) computes the PB, also excluding safety times used in DB calculations. Equation (9) calculates the CCB across sub-projects. The remaining three equations describe the influence of buffers on activity start times within and across sub-projects. Equation (10) captures the relationship between activity start times before and after the DB on a task chain. Equation (11) models the FB’s influence on activity start times before and after the FB on a non-critical chain. Equation (12) reflects the CCB’s influence on the start time of the drum activity between adjacent sub-projects.

### 3.2. Robustness measure

Robustness measures in scheduling models are essential for evaluating the stability and reliability of baseline schedules. In general, a robust baseline schedule should be developed prior to project implementation. It should be capable of absorbing unexpected disturbances during project execution. Research on robustness measures can be categorized into two types: direct and indirect measures.

Direct measures are derived from performance evaluations of schedule stability, such as the on-time completion rate, buffer consumption rate, and buffer overflow rate. These evaluations typically require prior knowledge of disturbance factors. However, assessing such factors in advance is challenging due to the uncertainties inherent in dynamic environments. This challenge is particularly pronounced in multi-project scheduling, where intense drum-resource competition and complex network structures exacerbate uncertainty.

Indirect measures are based on the quantitative evaluation of factors influencing schedule stability, such as time elasticity and resource elasticity. They do not require prior knowledge of how uncertain disturbance factors affect schedule performance. Accordingly, robustness can be evaluated more efficiently and with lower complexity using indirect measures. Therefore, an indirect measure is employed as the robustness measure in the proposed scheduling model.

Hazır et al. [9] employed the surplus time between a project’s deadline and its planned duration as an indirect robustness measure to estimate the probability of on-time completion. However, drum-resource interactions among sub-projects can significantly influence this probability. Specifically, conflicts over the drum resource may necessitate adjustments to sub-project schedules during conflict resolution, thereby altering the likelihood of on-time completion. Consequently, drum resource interactions must be incorporated into the robustness measure, as formulated in Equation (13).


$$RM=\text{1}\text{0}\text{0}\times \sum_{i}\left(\frac{{DRR}_{i}}{\sum_{i}{DRR}_{i}}\times \frac{\text{max}\left(D_{i}-s_{iJ_{i}-\text{1}}-{PB}_{i},\text{0}\right)}{s_{iJ_{i}-\text{1}}+{PB}_{i}}\right)$$ (13)

As shown in Equation (13), the robustness measure is defined as the sum of each sub-project’s robustness. Each sub-project’s robustness consists of two components: the first is a weight that estimates the relative reliance on the drum resource, and the second is the surplus-time ratio for the sub-project’s duration. The rationale for including these two components is as follows:

In the first component, the ratio of each sub-project’s Drum Resource Requirement (DRR) to the total DRR serves as a weight that estimates the relative reliance on the drum resource. The DRR represents the sum of all drum-resource requirements within a sub-project and serves as an overall estimate of the corresponding CCB and DBs, which are derived from the safety times of drum activities. Therefore, a sub-project with a larger DRR is associated with larger values of the CCB and DBs, resulting in greater time elasticity among sub-projects and a higher robustness weight.

In the second component, each sub-project’s surplus time is divided by its planned duration. A sub-project with greater surplus time results in greater robustness, as reflected by the proportional relationship in this ratio. Moreover, the robustness of each sub-project is safeguarded by a non-negativity truncation strategy. Specifically, if the planned duration exceeds the deadline, the robustness of the sub-project is set to zero using the expression $\text{max}\left(D_{i}-s_{iJ_{i}-\text{1}}-{PB}_{i},\text{0}\right)$.

## 4. Design of the enhanced discrete DE algorithm

### 4.1. Encoding and decoding schemes

**Fig 1:**
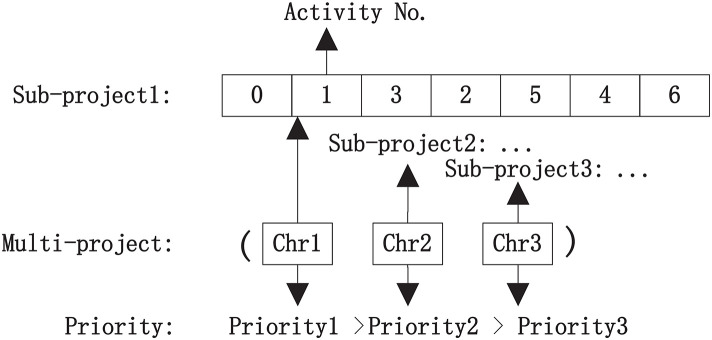
The set of chromosomes corresponding to a multi-project scheduling plan.

The traditional continuous DE algorithm represents individuals as priority vectors assigned to activities to encode and decode their processing sequences, where each priority is a real number that varies continuously within a defined interval. However, we use discrete encoding and decoding schemes in our study. Specifically, a set of chromosomes, composed of multiple distinct ordered chromosomes, corresponds to the encoding of a multi-project scheduling plan, with each chromosome representing a sub-project scheduling plan. Each gene in a chromosome represents an activity. The priority for the drum resource is determined by the sub-project unit tardiness cost. The sub-project with a greater unit tardiness cost has a higher priority. Fig 1 shows the encoding of the set of chromosomes corresponding to a multi-project scheduling plan. There are three sub-projects. The chromosomes corresponding to the three sub-projects are arranged in order of priority for the drum resource, from high to low. Different activities within a sub-project are arranged in a chromosome, subject to process precedence constraints. The feasible scheduling plan is then obtained through the decoding process.

In the decoding process, calculating the start and finish times for all activities in sub-projects is a necessary step. Different resource requirements should also be considered. Here, the most frequently used and most contested resource is regarded as the drum resource in the multi-project scheduling plan. Then, it is critical to identify the critical chain and non-critical chains within a sub-project scheduling plan, and to add PB at the end of the critical chain and FBs at the ends of the non-critical chains, respectively.

Moreover, it is necessary to identify the activities using the drum resource in each sub-project and to insert DBs before them. Based on the priority for drum-resource usage, for each new sub-project it is necessary to search both the late drum activity sets of implemented sub-projects and the early drum activity sets of implementing sub-projects [25]. The CCB should be inserted before the earliest conflicting drum activity in the early drum activity set and after the latest conflicting drum activity in the late drum activity set. Furthermore, the schedules of sub-projects should be adjusted, and the timing of drum-resource usage should be staggered based on the priority for the drum resource. The critical chain is then defined as the longest activity sequence that prevents the multi-project scheduling plan from finishing earlier. Finally, the robustness are computed as the decoding results based on the ‌serial scheduling generation scheme.

### 4.2. Elite-strategy-based hierarchical update mechanism

The traditional DE algorithm adopts a consistent differential evolution operation for all individuals in the population during each iteration. Thus, excellent individuals are treated identically to ordinary individuals, being subject to the same evolutionary operation, which results in low search efficiency and may miss better solutions. Therefore, an elite-strategy-based hierarchical update mechanism is proposed within the population. Specifically, the individuals are divided into two layers of different quality levels: the elite layer and the ordinary layer. The elite layer preserves the best individuals with better fitness in the population during each iteration. There are two reasons to design the elite layer. First, it facilitates maintaining and updating the dominant individuals during iterations. Second, it facilitates the population’s ability to conduct local search by using the historical information of dominant individuals. Moreover, the ordinary layer preserves the remaining individuals, which undergo only standard evolutionary operations during iterations. The individuals in the two layers must be updated promptly after each iteration of the algorithm.

### 4.3. Discrete differential evolution operators

The traditional DE algorithm uses differential evolution operators to search for scheduling results in the continuous solution space. During search iterations, the excellent individuals with better fitness are preserved, while the inferior individuals with poorer fitness are gradually eliminated. This process leads to the population gradually approaching the optimal solution. However, the project scheduling problem is a discrete optimization problem. A significant amount of time is consumed when transforming between the discrete problem space and the continuous solution space with this algorithm. Therefore, the quality and efficiency of the scheduling solutions are relatively low. To address this challenge, discrete differential evolution operators are required. Specifically, there are three types of evolutionary operators: crossover, mutation, and selection.

The first one is the mutation operator. Three individuals are randomly selected from the population, and a differential individual is produced by arithmetic calculations on the three individuals, influenced by a weight factor. The second one is the crossover operator. The differential individual and the original individual are crossed to generate a new individual with a crossover probability. The third one is the selection operator. In this step, the new individual is compared with the original individual, and a greedy process is applied to select the individual with the better fitness as the final result. Obviously, the final individual is produced collectively by the mutation, crossover, and selection operators. After the differential evolution operators are discretized, the final individual is updated as follows:


$$X_{i}^{k+\text{1}}=\text{Greedy}\left(X_{i}^{k},CR⊗\text{fh}\left(X_{i}^{k},F_{\text{2}}⊗\text{f}\left(F_{\text{1}}⊗{\text{f}}^{\prime }\left(X_{r\text{1}}^{k},X_{r\text{2}}^{k}\right),\;X_{r\text{3}}^{k}\right)\right)\right)$$ (14)

Here, $X_{i}^{k+\text{1}}$ represents the final individual produced by individual i in the (k+1)-th iteration; $X_{i}^{k}$ represents the final individual produced by individual i in the k**-**th iteration. This $X_{i}^{k}$ is also the original individual used for the evolutionary operation at the beginning of the (k+1)*-th* iteration; $X_{r\text{1}}^{k}$, $X_{r\text{2}}^{k}$, and $X_{r\text{3}}^{k}$ are three distinct individuals randomly selected from the results of the k**-**th iteration. These three are also different from $X_{i}^{k}$; $F_{\text{1}}$ and $F_{\text{2}}$ are weighting factors, and CR is the crossover probability. The variables $F_{\text{1}}$, $F_{\text{2}}$ and CR are all preset parameters within the interval [0, 1]. The discrete mutation, crossover, and selection operators are discussed below.

(1) ***Discrete mutation operator***

The traditional continuous mutation operator of the DE algorithm is based on two types of arithmetic calculations involving three individuals, for example, $A_{i}^{k+\text{1}}=X_{r\text{3}}^{k}+F*\left(X_{r\text{1}}^{k}-X_{r\text{2}}^{k}\right)$. Here, $A_{i}^{k+\text{1}}$ is the differential individual, and F is the weight factor, which lies in the range [0, 2]. The parameter F is used to reflect the degree of mutation. However, the traditional discrete mutation operator, as in the genetic algorithm (GA), introduces limited changes at specific gene loci on a chromosome, while the traditional discrete crossover operator affects evolutionary results by recombining genes at different loci across chromosomes. To retain the characteristics of the continuous mutation operator in the DE algorithm, such as the two types of arithmetic calculations and varying degrees of evolutionary results, a single-point crossover f and a two-point crossover ${\text{f}}^{\prime }$ are used in the discrete mutation operator of our algorithm. The specific formula is ${A_{i}^{k+\text{1}}=F}_{\text{2}}⊗\text{f}\left(F_{\text{1}}⊗{\text{f}}^{\prime }\left(X_{r\text{1}}^{k},X_{r\text{2}}^{k}\right),X_{r\text{3}}^{k}\right)$, and its implementation is shown in Algorithm 1.

Algorithm 1. **The discrete mutation operator.**

**Input:** Individuals $X_{r\text{1}}^{k},X_{r\text{2}}^{k},andX_{r\text{3}}^{k},weightingfactorsF_{\text{1}}andF_{\text{2}}$;

**Output:** Differential individual $A_{i}^{k+\text{1}}$;

1. Generate a random number, and store it in ${rand}_{\text{1}}$;

2. **if** ${rand}_{\text{1}}\leq F_{\text{1}}$** then**

3.    $A_{i(\text{1})}^{k+\text{1}}={\text{f}}^{\prime }\left(X_{r\text{1}}^{k},X_{r\text{2}}^{k}\right)$; % two-point crossover

4. **else**

5.    $A_{i(\text{1})}^{k+\text{1}}=X_{r\text{1}}^{k}$;

**6. end if**

**7.** Generate a random number, and save it in ${rand}_{\text{2}}$;

8. **if** ${rand}_{\text{2}}\leq F_{\text{2}}$** then**

9.    $A_{i(\text{2})}^{k+\text{1}}=\text{f}\left(A_{i(\text{1})}^{k+\text{1}},X_{r\text{3}}^{k}\right)$; % single-point crossover

10. **else**

11.    $A_{i(\text{2})}^{k+\text{1}}=A_{i(\text{1})}^{k+\text{1}}$;

**12.end if**

13.$A_{i}^{k+\text{1}}=A_{i(\text{2})}^{k+\text{1}}$;

14.**return** $A_{i}^{k+\text{1}}$

Algorithm 1 shows that the two-point crossover ${\text{f}}^{\prime }$ is used to replace the subtraction $X_{r\text{1}}^{k}-X_{r\text{2}}^{k}$ in the continuous mutation operator. This is done because the two-point crossover can lead to a strong differential result, which is similar to the subtraction with the weighting factor F in the continuous mutation operator. The result is saved as $A_{i(\text{1})}^{k+\text{1}}$ with a weighting factor $F_{\text{1}}$, using the formula: $A_{i(\text{1})}^{k+\text{1}}=F_{\text{1}}⊗{\text{f}}^{\prime }\left(X_{r\text{1}}^{k},X_{r\text{2}}^{k}\right)$. Specifically, a random number ${rand}_{\text{1}}$ is generated and compared with the weighting factor $F_{\text{1}}$. If ${rand}_{\text{1}}$ is less than $F_{\text{1}}$, a two-point crossover ${\text{f}}^{\prime }$ is performed; otherwise, $X_{r\text{1}}^{k}$ remains unchanged. A single-point crossover f is adopted to replace the addition $A_{i(\text{1})}^{k+\text{1}}+X_{r\text{3}}^{k}$ in the continuous mutation operator. This is done because the single-point crossover can lead to a weak differential result, which is similar to the addition process. The result is saved as $A_{i(\text{2})}^{k+\text{1}}$ with a weighting factor $F_{\text{2}}$, using the formula: $A_{i(\text{2})}^{k+\text{1}}=F_{\text{2}}⊗\text{f}\left(A_{i(\text{1})}^{k+\text{1}},X_{r\text{3}}^{k}\right)$. Specifically, a random number ${rand}_{\text{2}}$ is generated and compared with the weighting factor $F_{\text{2}}$. If ${rand}_{\text{2}}$ is less than $F_{\text{2}}$, a single-point crossover f is performed; otherwise, $A_{i(\text{1})}^{k+\text{1}}$ remains unchanged. After these two steps, the differential individual $A_{i}^{k+\text{1}}$ is produced.

(2) Discrete crossover operator.

In the traditional continuous crossover operator, each dimension of the new individual is randomly selected from either the corresponding dimension of the differential individual $A_{i}^{k+\text{1}}$ or the original individual $X_{i}^{k}$ with the crossover probability CR. Meanwhile, at least one value in the new individual is required to be equal to that of the differential individual $A_{i}^{k+\text{1}}$. Obviously, there are two sources for constructing the new individual, each corresponding to distinct options with limited degrees of change. Therefore, the traditional discrete single-point crossover f and single-point mutation h in GA are adopted to discretize the continuous crossover operator under different types of options with limited changes. The new individual is saved as $B_{i}^{k+\text{1}}$, with the formula $B_{i}^{k+\text{1}}=CR⊗\text{fh}\left(X_{i}^{k},A_{i}^{k+\text{1}}\right)$. The implementation process is presented in Algorithm 2.

Algorithm 2. **The discrete crossover operator.**

**Input:** Original individual $X_{i}^{k}$, differential individual $A_{i}^{k+\text{1}}$, crossover probability CR;

**Output:** New individual $B_{i}^{k+\text{1}}$;

1. Generate a random number, and store it in ${rand}_{\text{3}}$;

2. **if** ${rand}_{\text{3}}\leq CR$** then**

3.    $B_{i}^{k+\text{1}}=\text{f}\left(X_{i}^{k},A_{i}^{k+\text{1}}\right)$; % single-point crossover

4. **else**

5.    $B_{i}^{k+\text{1}}=\text{h}\left(X_{i}^{k}\right)$; % single-point mutation

**6. end if**

7. **return** $B_{i}^{k+\text{1}}$

According to Algorithm 2, the discrete crossover operation proceeds as follows: First, a random number ${rand}_{\text{3}}$ is generated and compared with the crossover probability CR. Second, if ${rand}_{\text{3}}$ is less than CR, the single-point crossover $B_{i}^{k+\text{1}}=\text{f}\left(X_{i}^{k},A_{i}^{k+\text{1}}\right)$ is performed; otherwise, the single-point mutation $B_{i}^{k+\text{1}}=\text{h}\left(X_{i}^{k}\right)$ is performed. Finally, the new individual $B_{i}^{k+\text{1}}$ is obtained.

(3) Discrete selection operator.

As in the traditional continuous DE algorithm, a greedy selection is also performed by the discrete selection operator in our algorithm, based on the fitness of the original individual $X_{i}^{k}$ and the new individual $B_{i}^{k+\text{1}}$. The individual with better fitness is selected as the final result of the (k+1)-th iteration, and saved as $X_{i}^{k+\text{1}}$ with the formula $X_{i}^{k+\text{1}}=Greedy\left(X_{i}^{k},B_{i}^{k+\text{1}}\right)$.

### 4.4. Hill-climbing local search

In the traditional DE algorithm, differential evolution operators are applied in parallel to all individuals in the population. When convergence to the optimum is slow after numerous iterations, global searches across all individuals yield little improvement. Meanwhile, if the information from previously identified high-quality individuals cannot be effectively utilized, the search process becomes inefficient. Thus, it is necessary to leverage the information from previously identified high-quality individuals and to adopt a local search strategy.

However, two challenges arise. First, unlike global parallel search, local search typically employs different operators to enhance population diversity and help the algorithm escape from local optima. Second, local search is computationally intensive. If too many individuals are involved, the algorithm’s overall efficiency may be significantly reduced; conversely, if too few are involved, the effectiveness of the search will be limited. The Hill-Climbing (HC) algorithm serves as a local search method. It quickly and effectively converges to a local optimum from a given point in the solution space [26]. Moreover, a hierarchical initialization mechanism is adopted. The elite layer preserves the key information of high-quality individuals. These individuals also exhibit superior fitness and high sensitivity, thereby enhancing population diversity and improving search efficiency. Therefore, we propose a hierarchical local search strategy based on the HC algorithm, targeting individuals in the elite layer. See Algorithm 3 for further details.

Algorithm 3. **The hierarchical local search process based on the HC algorithm.**

**Input:** Elite layer individual array elites, elite layer individual number elitesNum;

**Output:** Updated elite layer individual array elites;

1. **for** i=1𝐭𝐨elitesNum **do** % local search on all the individuals in elite layer

2.  Compute the fitness f corresponding to the activity series actSeries of elites[i];

3.  Select a drum activity (with its index of j) randomly from actSeries;

4.  Find the maximum direct predecessor index (prej) of activity j;

5.  Find the minimum direct successor index (succj) of activity j;

6.  **for** k=prej+1𝐭𝐨succj−1 **do** % neighborhood search between prej+1 and succj−1

7.   Change the activities at positions k and j, save the new job series as $actSeries^{\prime };$

8.   Compute the new fitness $f^{\prime }$ corresponding to $actSeries^{\prime }$;

9.   **if** $f^{\prime }$ is better than f **then**

10.   Update the job series of elites[i] to $actSeries^{\prime }$; % obtain a better individual

**11.   end if**

**12. end for**

**13. end for**

14. **return** elites % the updated elites

As shown in Algorithm 3, the local search process for all individuals in the elite layer proceeds as follows. Specifically, the search is performed for each individual. First, a drum activity (with index j) is randomly selected from the activity sequence array actSeries of the elite layer individual elites[i]. Second, the maximum direct predecessor (with index prej) of the drum activity is identified. Third, the minimum direct successor (with index succj) of the drum activity is identified. Fourth, all activities between indices prej+1 and succj−1 in the activity sequence array actSeries are traversed. Each traversed activity is then exchanged with the drum activity j, ensuring that the resulting sequence adheres to the original activity precedence constraints. A new activity sequence array $actSeries^{\prime }$ is generated, and its corresponding fitness is calculated. If the new fitness is better than the original, the original array is replaced with $actSeries^{\prime }$. Then, the activity sequence array of elites[i] is updated accordingly. Finally, the updated elites are returned after the local search for all individuals is completed.

### 4.5 Overall framework

In summary, the framework of the enhanced discrete DE algorithm is presented as follows:

**Step 1:** Population initialization. Assign initial values to the algorithm parameters, including population size N, maximum number of iterations M, number of elite individuals elitesNum, and other relevant factors. According to the encoding/decoding schemes described in Section 4.1 and the hierarchical initialization method in Section 4.2, generate N encoded groups representing chromosome combinations of multi-project scheduling solutions. Decode and evaluate the fitness of these groups; select individuals with the best fitness for the elite layer, and assign the remaining individuals to the ordinary layer. The variable bestIcon denotes the global best individual, initialized as the chromosome combination with the highest fitness in the population.

**Step 2:** Check whether the termination condition is satisfied. If satisfied, terminate the iterations and proceed to Step 5; otherwise, apply the differential evolution operators (mutation, crossover, and selection) to all individuals in the population, as described in Section 4.3.

**Step 3:** Perform hierarchical local search based on the HC algorithm for individuals in the elite layer, as described in Section 4.4.

**Step 4:** Update the individuals in both the elite and ordinary layers; set bestIcon to the global best individual; and return to Step 2.

**Step 5:** Output the global best individual, bestIcon, as the final robust scheduling solution.

Based on the algorithmic framework above, the time complexity can be computed. Because the ‌serial scheduling generation scheme is used in the encoding/decoding scheme, the per-individual cost is $\Theta \left(n^{2}m\right)$ [27]. Accordingly, the overall time complexity is $\Theta \left(n^{2}mMN\right)$ without local search. When local search is employed, the overall time complexity is $\Theta \left(n^{3}mMN\right)$ in the worst case. Here, n is the total number of activities ($n=\sum_{i}J_{i}$); m is the number of resource types (${m=K}_{l}+\text{1}$); and N and M are the population size and the maximum number of iterations, respectively.

## 5. Experimental setup and validation

### 5.1. Test case generation and parameter settings

Two sets of experiments were conducted to validate the proposed methodology. The first set aimed to assess the effectiveness of the enhanced discrete DE algorithm (see Section 5.3). Several theoretical multi-project scheduling instances of varying scales were tested, and the results obtained by our enhanced discrete DE algorithm were compared with those from other heuristic algorithms. The second set aimed to validate the effectiveness of the robustness measure (see Section 5.4). An industrial case was used for testing. The results obtained under the maximum robustness objective were compared with those under other objectives across various uncertainty levels. Details of the two test sets are provided below.

In the absence of standardized multi-project scheduling benchmarks for algorithm evaluation, this study employed a project instance generator (ProGen) to generate sub-project scheduling instances. Subsequently, multi-project scheduling instances were constructed by combining distinct sub-project instances. Four types of instances were considered: 3-sub-project portfolios (PJS3−1 and PJS3−2), 5-sub-project portfolios (PJS5−1 and PJS5−2), 10-sub-project portfolios (PJS10−1 and PJS10−2), and 20-sub-project portfolios (PJS20−1 and PJS20−2). These instances considered only renewable resources, including local resources within sub-projects and a single global resource shared among the sub-projects. Since the global resource was derived from the most contested local resource type, its capacity was set to the maximum of that resource’s available capacities across the sub-projects.

A practical multi-project scheduling example from Qing’an Corporation was used to validate the proposed robustness measure. The corporation is one of the largest manufacturers of aviation-related mechanical equipment in China. The corporation manages thousands of projects annually. Most projects within the corporation involve intense resource contention, and some must be scheduled in parallel. Investigations indicated that multi-project management at Qing’an Corporation was characterized by high uncertainty; consequently, scheduling plans were difficult to implement effectively, which resulted in significant project delays. Therefore, a representative multi-project scheduling example comprising three sub-projects was selected (see Table 3).

**Table 3:** Multi-project scheduling example from Qing’an Corporation.

Project ID	Activity ID	Successors	Duration	Resource Requirement				Due date	Resource Supply			
				r1		r2	r3	r4	R1	R2	R3	R4
0	0	1,2	0	0	0	0	0	150	4	12	24	2
	1	3	23	1	5	7	1					
	2	4	36	1	5	7	1					
	3	4,5,6	1	0	0	0	2					
	4	7	31	2	6	12	1					
	5	7	57	2	6	12	1					
	6	7	102	2	6	12	1					
	7	8	1	0	0	0	2					
	8	NULL	0	0	0	0	0					
1	0	1,2	0	0	0	0	0	85	8	0	16	1
	1	3	16	4	0	8	0					
	2	4	33	4	0	8	0					
	3	4,5,6	1	1	0	0	1					
	4	7	23	4	0	8	0					
	5	7	35	4	0	8	0					
	6	7	50	4	0	8	0					
	7	8	1	1	0	0	1					
	8	NULL	0	0	0	0	0					
2	0	1,2	0	0	0	0	0	106	12	5	12	1
	1	3	21	3	2	5	0					
	2	4	23	4	2	5	0					
	3	4,5,6	1	1	0	0	1					
	4	7	19	4	3	6	0					
	5	7	51	6	2	6	0					
	6	7	72	6	2	6	0					
	7	8	1	1	0	0	1					
	8	NULL	0	0	0	0	0					

Additionally, the parameters used in our experiment were not only based on existing studies [15,24], but were also validated and adjusted to the specific characteristics of the problem addressed in this study. Specifically, the DE algorithm used a population of 20 individuals, including 5 elite individuals, and was executed for up to 50 iterations. The weighting factors $F_{\text{1}}$ and $F_{\text{2}}$ were set to 0.9 and 0.45, respectively, and the crossover probability (CR) to 0.9. Other relevant parameters are listed in Table 4. To apply CCM to the scheduling models, the activities’ most likely durations and safety times were computed as described in Section 5.2.

**Table 4:** Experimental parameter settings.

Individual number	20	Multi-project scale	3、5、10、20
Elite number	5	Non-dummy activity number in sub-projects	[5,8]
Maximum iteration number	30	Activity execution mode number	1
Test times of each test case	30	Maximum activity duration	10
F_1_	0.9	Local renewable resource types	2
F_2_	0.45	Global renewable resource types	1
CR	0.9	Resource maximum supply	10

### 5.2. Calculation of the most likely duration and safety time

In this study, multi-project scheduling project is modeled under uncertainty, where sub-project activity durations are subject to external disturbances. To accurately capture the stochastic nature of activity durations, a probability distribution is specified. Typically, activity durations are strictly positive, bounded below by a theoretical minimum, and characterized by asymmetry: a minority are shorter than the most likely value, whereas most are longer, with some experiencing significant delays. The lognormal distribution, which embodies these characteristics, is therefore adopted to model uncertain activity durations.

Specifically, the uncertain duration of activity $a_{ij}$ in sub-project $P_{i}$ is modeled as ${ad}_{ij}\sim \text{Lognormal}\left(\mu_{ij},\sigma_{ij}\right)$, where ${ad}_{ij}$ denotes the random variable representing the activity duration. Here, $\mu_{ij}$ and $\sigma_{ij}$ represent the mean and standard deviation of the natural logarithm of the duration, respectively, while the most likely duration $d_{ij}$ equals the mode of the distribution. The coefficient of variation (CV), defined as $cv=\sigma_{ij}/\mu_{ij}$, quantifies the relative variability of activity durations. A higher CV reflects greater uncertainty in scheduling estimates.

In general, project scheduling uncertainty is moderate, and activity duration estimates tend to be conservative. Accordingly, Section 5.3 uses a CV of 0.3 and assumes that activity durations correspond to a 95% completion probability. Based on these settings, the most likely durations and safety times were determined by inverting the lognormal cumulative distribution function. Moreover, to assess the proposed robustness measure under different uncertainty conditions, nine risk levels were examined in Section 5.4, with CV values ranging from 0.1 to 0.9. Considering that frequent project delays at Qing’an Corporation stem partly from underestimated durations, activity durations in this case are assumed to correspond to an 80% completion probability.

### 5.3. Validation of the enhanced discrete DE algorithm

To evaluate the effectiveness of the enhanced discrete DE algorithm, eight instances were examined. Two sets of experiments were conducted here: the first set compared the enhanced discrete DE with other algorithms, such as the genetic algorithm (GA) and particle swarm optimization (PSO); the second set focused on an ablation analysis of each new component proposed in our algorithm, where the enhanced discrete DE (with the enhanced local search) was compared with the discrete DE algorithm and the traditional continuous DE algorithm, both without the enhanced local search. Each instance was solved ten times, and the scheduling results were collected. The average robustness (AR) and average computation time (ACT) for the two experiments are reported in Tables 5 and 6, respectively.

**Table 5:** Comparison of results obtained by different heuristic algorithms.

Instances	Enhanced discrete DE		GA		PSO	
	AR	ACT	AR	ACT	AR	ACT
PJS3−1	6.3	44.9	6.1	27.7	6.2	27.1
PJS3−2	10.1	47.7	10.0	26.0	9.9	30.4
PJS5−1	26.6	128.8	26.5	63.7	26.6	103.4
PJS5−2	0.5	105.3	0.4	54.2	0.3	66.7
PJS10−1	65.4	288.7	61.9	178.0	65.2	139.3
PJS10−2	17.9	277.6	17.9	154.4	16.5	218.6
PJS20−1	20.1	759.6	19.3	480.0	19.4	645.2
PJS20−2	24.8	695.8	24.5	503.1	24.7	610.9

**Table 6:** Comparison of results obtained by different DE variants.

Instances	Enhanced discrete DE		Discrete DE		Continuous DE	
	AR	ACT	AR	ACT	AR	ACT
PJS3−1	6.3	44.9	6.1	32.9	5.9	39.6
PJS3−2	10.1	47.7	10.0	35.9	9.0	51.4
PJS5−1	26.6	128.8	26.6	94.3	26.6	119.3
PJS5−2	0.5	105.3	0.4	102.0	0.4	94.2
PJS10−1	65.4	288.7	61.7	231.4	60.5	219
PJS10−2	17.9	277.6	17.6	246.6	17.3	249.9
PJS20−1	20.1	759.6	19.0	693.4	20.1	654.4
PJS20−2	24.8	695.8	24.3	685.6	24.0	648.1

As shown in Table 5, the enhanced discrete DE algorithm achieves the highest average robustness in most cases, but it also requires the longest average computation time. Compared with GA, PSO yields similar average robustness but generally requires more computation time. Based on the average robustness of different algorithms, two sets of statistical tests were conducted to verify the significance of the differences.

We applied the Friedman test to the eight instances. It revealed a significant overall difference among the algorithms (χ²(2)=10.07, Kendall’s W=0.63). Additionally, we used the Wilcoxon signed-rank test with Holm correction to compare the enhanced discrete DE with GA and PSO. The results also indicated significant pairwise differences. Specifically, p=0.04 for enhanced discrete DE vs. GA, and p=0.04 for enhanced discrete DE vs. PSO. Furthermore, the median paired differences were 0.15 and 0.20, respectively, with the rank-biserial correlation r=1.00 for both. These results demonstrate that our enhanced discrete DE outperforms GA and PSO on most instances.

**Fig 2:**
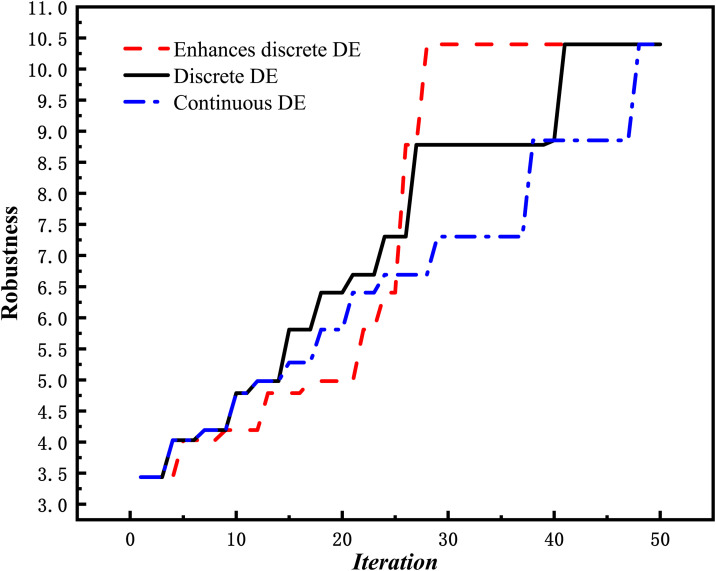
Comparison of robustness during iterations based on different DE variants.

As shown in Table 6, the enhanced discrete DE (with local search) still perform best in terms of average robustness but requires the longest average computation time. Compared with the traditional continuous DE, the discrete DE algorithm achieves higher average robustness, although its average computation time has no clear advantage over that of the traditional continuous DE algorithm. To further assess the convergence, a line chart for instance PJS3−2 showing the iteration-wise performance of these DE variants is provided in Fig 2.

From Fig 2, we can see that the enhanced discrete DE converges to its global best value by the 30th iteration. Although the other algorithms appear to converge much fast in the early stages, they are prone to falling into the local optima due to extended plateau. Moreover, the discrete DE behaves much better in terms of average robustness than the traditional continuous DE, with a slightly faster convergence speed than that of the traditional continuous DE. The reasons for these results are threefold.

First, the discrete DE algorithm offers limited savings in computation time. Although it avoids transformations between the discrete problem space and the continuous solution space, its evolutionary operators are more complex than those in the continuous version. Specifically, discrete operators involve exchanging or recombining activity sequences according to predefined rules, whereas continuous operators rely primarily on simple arithmetic operations, such as vector addition and subtraction. This added complexity offsets the time saved by avoiding these transformations and may further increase computational effort.

Second, discrete evolutionary operators provide greater flexibility in the search process than their continuous counterparts. They enable the direct generation of new feasible individuals by exchanging or reordering activity sequences while preserving predefined precedence constraints. In contrast, continuous evolutionary operators modify the priority parameters for individual activities. However, a single priority value per activity is insufficient to uniquely determine the resulting processing sequence. This often results in inefficient search outcomes, with many solutions violating the predefined precedence constraints.

Third, the enhanced local search substantially improves solution quality. The discrete DE algorithm without local search generates new individuals via parallel evolutionary operations, but it exhibits limited local search capability and may miss superior solutions. In contrast, the enhanced local search proposed in this study detects stagnation and intensifies a neighborhood search around the current best individual using a hill-climbing procedure, thereby improving solution quality in the vicinity of local optima. On average across the eight instances, the enhanced discrete DE algorithm improves robustness by more than 3.3% relative to the overall mean of the benchmark algorithms.

### 5.4. Validation of the robustness measure

To validate the robustness measure proposed in this study, we compare the stability of scheduling plans derived from different strategies using a practical multi-project scheduling example. First, we solve the example by maximizing the robustness objective with CCM. The resulting robustness value is 14.08. We also solve the example by minimizing the multi-project duration objective, both with and without CCM. The multi-project durations are 312 and 330 days, respectively. To assess the stability of these plans in a dynamic environment, we use different CVs to represent uncertainty levels. For each plan at a given CV level, ten Monte Carlo simulation runs are conducted, each comprising 1000 replications. The averages of the on-time completion rate (AOTCR), buffer consumption rate (ABCR), buffer overflow rate (ABOR), and multi-project duration (AMPD) are recorded in Tables 7. Since the plan without CCM contains no buffers, ABCR and ABOR are not reported in Table 9.

**Table 7:** Simulation results for maximizing the robustness objective with CCM.

Uncertain scheduling simulation results	The CV of activity durations								
	0.1	0.2	0.3	0.4	0.5	0.6	0.7	0.8	0.9
**AOTCR (%)**	100.00	98.87	95.60	92.03	87.46	82.3	81.57	75.23	74.73
**ABOR (%)**	0.33	4.17	9.08	12.62	15.63	19.12	19.50	22.31	23.69
**ABCR (%)**	1.51	10.67	34.23	56.82	93.25	159.65	176.68	261.88	268.48
**AMPD**	255.94	258.17	261.24	264.38	262.90	273.46	275.03	280.87	283.98

**Table 8:** Simulation results for minimizing the multi-project duration objective with CCM.

Uncertain scheduling simulation results	The CV of activity durations								
	0.1	0.2	0.3	0.4	0.5	0.6	0.7	0.8	0.9
**AOTCR (%)**	100.00	98.53	95.47	90.83	84.07	79.23	76.23	72.10	71.97
**ABOR (%)**	0.23	4.54	9.1	14.91	20.16	24.05	24.76	27.14	25.64
**ABCR (%)**	1.52	13.03	35.45	76.74	126.00	191.91	250.14	315.20	324.68
**AMPD**	256.26	263.78	267.79	274.34	285.43	294.48	296.36	302.67	304.38

**Table 9:** Simulation results for minimizing the multi-project duration objective without CCM.

Uncertain scheduling simulation results	The CV of activity durations								
	0.1	0.2	0.3	0.4	0.5	0.6	0.7	0.8	0.9
**AOTCR (%)**	99.97	97.50	91.47	83.40	77.37	70.20	70.04	64.63	62.06
**AMPD**	280.64	287.06	295.22	303.30	310.45	320.06	321.57	329.51	343.39

**Fig 3:**
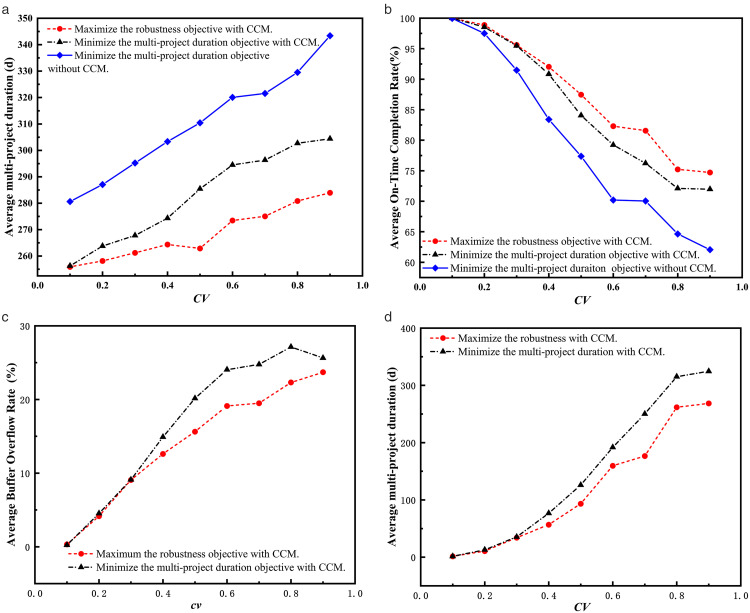
(a-d). Comparison of simulation results under different strategies. From Fig 3a–d, we observe that the simulation results of maximizing the robustness objective with CCM achieve the best performance in terms of average multi-project duration, average on-time completion rate, average buffer overflow rate, and average buffer consumption rate. Meanwhile, the simulation results of minimizing the multi-project duration objective with CCM are worse than those from maximizing the robustness objective with CCM, but better than those from minimizing the multi-project duration objective without CCM. There are two main reasons for this. On the one hand, CCM not only provides necessary buffers to increase schedule flexibility, but also reduces the safety times embedded in activity durations. Thus, the simulation results of minimizing the multi-project duration objective with CCM are better than those of minimizing the multi-project duration objective without CCM. On the other hand, the robustness measure proposed in this study not only considers the time surplus within each sub-project but also balances the drum resource requirements across sub-projects. This measure gives sub-projects with greater drum resource requirements or later due dates higher priority in accessing contested resources, leading to more stable execution.

As shown in Tables 7–9, increasing risk levels lead to a gradual decrease in the average on-time completion rate, along with gradual increases in the average buffer consumption rate, average buffer overflow rate, and average multi-project duration. This is because the variability of activity durations increases as the CV increases, thereby increasing the likelihood of activity delays and resulting in greater buffer consumption and overflow, as well as longer multi-project durations and reduced on-time completion rates. To further analyze the differences in the simulation results among these scheduling plans, line charts are plotted for the different optimization strategies, as shown in Fig 3a–d).

## 6. Conclusions

To address uncertainties in dynamic environments, the CCM is adopted in multi-project scheduling to improve robustness. Specifically, different types of buffers are incorporated into the multi-project scheduling model, with a newly designed robustness measure as the optimization objective. Meanwhile, an enhanced discrete DE algorithm is proposed to solve this problem. Two sets of experiments were conducted to validate the effectiveness of the algorithm and the robustness measure. The results show that: (1) Although the discrete DE algorithm requires more computation time, it achieves better robustness, with the average across the eight instances being 3.3% higher than the overall mean of the benchmark algorithms. (2) Applying the CCM improves the stability of the scheduling plan and shortens the multi-project duration, while the proposed robustness measure further strengthens stability and reduces buffer consumption and overflow.

The contributions are twofold. First, a robustness measure is proposed to evaluate the stability of the multi-project scheduling plan, taking into account both time elasticity within and among sub-projects. Second, the enhanced discrete DE algorithm not only discretizes the evolutionary operators and the encoding-decoding strategy to reduce transformations between the continuous solution space and the discrete problem space, but also uses a hill-climbing algorithm to enhance local search.

This paper addresses the impact of uncertainty on project duration in multi-project scheduling, thereby enhancing the stability of project implementation. However, this paper focuses on a single robustness objective. In management practice, project managers always consider multiple objectives simultaneously, such as robustness, duration, and cost. These objectives often conflict, further increasing the complexity of the optimization process. For example, enhancing robustness or reducing cost may extend the project duration. Therefore, future work will focus on multi-objective optimization in multi-project scheduling, investigating trade-offs between robustness and other objectives.

## Supporting information

S1 Data Testcases and code. (RAR)
